# Antimicrobial activity of a C-terminal peptide from human extracellular superoxide dismutase

**DOI:** 10.1186/1756-0500-2-136

**Published:** 2009-07-15

**Authors:** Mukesh Pasupuleti, Mina Davoudi, Martin Malmsten, Artur Schmidtchen

**Affiliations:** 1 Division of Dermatology and Venereology, Department of Clinical Sciences, Biomedical Center B14, Lund University, Tornavägen 10, SE-22184 Lund, Sweden; 2 Department of Pharmacy, P.O. Box 580, Uppsala University, SE-75123 Uppsala, Sweden

## Abstract

**Background:**

Antimicrobial peptides (AMP) are important effectors of the innate immune system. Although there is increasing evidence that AMPs influence bacteria in a multitude of ways, bacterial wall rupture plays the pivotal role in the bactericidal action of AMPs. Structurally, AMPs share many similarities with endogenous heparin-binding peptides with respect to secondary structure, cationicity, and amphipathicity.

**Findings:**

In this study, we show that RQA21 (RQAREHSERKKRRRESECKAA), a cationic and hydrophilic heparin-binding peptide corresponding to the C-terminal region of extracellular superoxide dismutase (SOD), exerts antimicrobial activity against *Escherichia coli*, *Pseudomonas aeruginosa*, *Staphylococcus aureus*, *Bacillus subtilis *and *Candida albicans*. The peptide was also found to induce membrane leakage of negatively charged liposomes. However, its antibacterial effects were abrogated in physiological salt conditions as well as in plasma.

**Conclusion:**

The results provide further evidence that heparin-binding peptide regions are multifunctional, but also illustrate that cationicity alone is not sufficient for AMP function at physiological conditions. However, our observation, apart from providing a link between heparin-binding peptides and AMPs, raises the hypothesis that proteolytically generated C-terminal SOD-derived peptides could interact with, and possibly counteract bacteria. Further studies are therefore merited to study a possible role of SOD in host defence.

## Background

Antimicrobial peptides (AMP) are short cationic peptides widely distributed at biological surfaces prone to infection, playing important roles in innate immunity [1]. At present at least 900 different AMP have been discovered . Many AMPs are characterized by an amphipathic structure, where clusters of hydrophobic and cationic amino acids are spatially organized in sectors within the molecules. For example, AMPs comprise linear peptides, many of which may adopt α-helical and amphipathic conformation upon bacterial binding, peptides forming cysteine-linked antiparallel β-sheets, as well as cysteine-constrained loop structures. AMPs may also, however, be found among peptides not displaying such ordered structures as long as these are characterized by an over-representation of certain amino acids [1]. Considering the role of peptide secondary structure and amphphilicity, as well as the composition of bacterial membranes, AMP function has been thought to involve direct binding to the lipid bilayer, and the interaction with bacterial membranes is a prerequisite for AMP function. However, the modes of action of AMPs on their target bacteria are complex, and can be divided into membrane disruptive and non-membrane disruptive [1,2]. Nevertheless, the lack of a specific molecular microbial target minimizes the risk of resistance development and thus AMPs may have therapeutic potential [3].

Superoxide dismutase (SOD) is a metalloenzyme that catalyzes the dismutation of superoxide radicals [4]. Mammalian cell produce two different types of SOD, one intracellular and one extracellular form. Extracellular superoxide dismutase (EC-SOD) is the major form (>90% of total), present in all extracellular fluids, including plasma, lymph, synovial fluid, brain grey matter, liver, kidney, and spleen [4,5]. Interestingly EC-SOD, via its C-terminal domain, binds to glycosaminoglycans, such as heparan sulfate and heparin [6-9]. We have previously shown that cationic AMPs, including cathelicidins and defensins, may interact with negatively charged glycosaminoglycans (GAGs) like heparin [10]. Conversely, we have demonstrated that heparin-binding motifs [11,12], as well as peptide sequences of endogenous proteins including peptides derived from complement [13,14], human kininogen [15], matrix proteins [16], growth factors [17], and histidine-rich glycoprotein [18,19], all exhibit antimicrobial effects, thus acting as "classical" peptides and proteins of innate immunity. These observations prompted us investigate possible antimicrobial effects of the C-terminal heparin binding domain of EC-SOD. We here identify such an activity, and define it in terms of action on various microbes and on eukaryotic cells, as well as the influence on these effects of plasma and the ionic environment.

## Materials

### Peptides and bacteria

The peptides RQA21 (RQAREHSERKKRRRESECKAA) and LL-37 (LLGDFFRKSKEKIGKEFKRIVQRIKDFLRNLVPRTES) were from Innovagen AB, Lund, Sweden. The purity (>95%) and molecular weight of these peptides was confirmed by mass spectral analysis (MALDI.TOF Voyager). *Escherichia coli *ATCC 25922 and clinical isolate 37.4, *Pseudomonas aeruginosa *ATCC 27853 and clinical isolate 27.5, *Staphylococcus aureus *ATCC 29213 and clinical isolate 4, *Bacillus subtilis *ATCC 6633, and *Candida albicans *ATCC 90028 were all obtained from the Department of Clinical Bacteriology at Lund University Hospital.

### Radial diffusion assay

This was performed according to procedures described previously [20]. 6 μl of test sample (100 μM) was added to each well and for comparison, LL-37 was used.

### Viable-count analysis

*E. coli *37.4 were subjected to peptides at 30 and 60 μM in 10 mM Tris, with or without 0.15 M NaCl as previously described [20].

### Hemolysis and LDH assay

These assays were preformed according to procedures described previously [17,20].

### Liposome preparation and leakage assay

The liposomes investigated were anionic (DOPE/DOPG 75/25 mol/mol). DOPG (1,2-Dioleoyl-*sn*-Glycero-3-Phosphoglycerol, monosodium salt) and DOPE (1,2-dioleoyl-*sn*-Glycero-3-phoshoethanolamine) were from Avanti Polar Lipids (Alabaster, USA) and of >99% purity. Leakage assay was performed according to previously published procedures [20]. A SPEX-fluorolog 1650 0.22-m double spectrometer (SPEX Industries, Edison, USA) was used for the liposome leakage assay. Measurements were performed in triplicate at 37°C.

## Results

**Figure 1:**
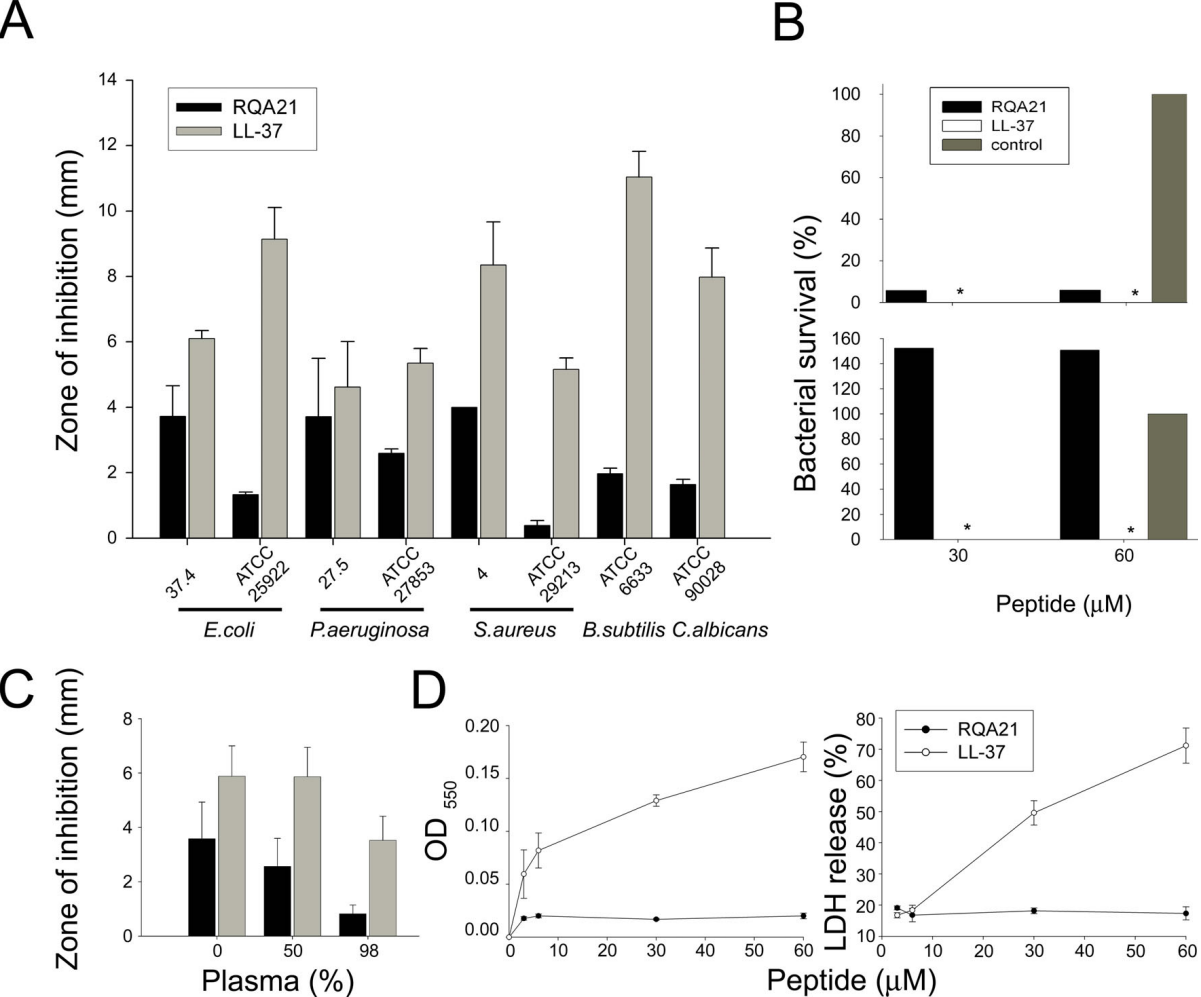
**Activities of selected peptides**. **(A) **Antimicrobial activity of RQA21 and LL37 peptides (at 100 μM in RDA) against the indicated microbes. Bacteria (4 × 10^6 ^cfu) or *C. albicans *ATCC 90028 (1 × 10^5^) was inoculated in 0.1% TSB agarose gel. The zones of clearance correspond to the inhibitory effect of each peptide (6 μl at 100 μM) after incubation at 37°C for 18–24 h (mean values are presented, n = 3). **(B) **2 × 10^6 ^colony-forming units/ml of *E. coli *37.4 bacteria were incubated in 50 μl with peptides at 30 and 60 μM for 2 hours followed by plating on TSB agar plates and cfu were determined. Upper panel; in 10 mM Tris, pH 7.4, 5 mM glucose, lower panel; the same buffer with 0.15 M NaCl. **(C) **Antimicrobial activity of RQA21 and LL37 peptides (at 100 μM in RDA) against *E. coli *37.4 in the presence of citrate plasma at the indicated concentrations. **(D) **Left panel; Analysis of hemolytic effects of RQA21 peptide and comparison with LL-37. The cells were incubated with peptides at the indicated concentrations (x-axis). 2% Triton X-100 (Sigma-Aldrich) served as positive control. The absorbance of hemoglobin release was measured at λ 550 nm and is expressed as % of Triton X-100 induced hemolysis (y-axis) (n = 3, mean values and SD is indicated). Right panel; HaCaT keratinocytes were subjected to RQA21 and LL-37. Cell permeabilizing effects were measured by the LDH based TOX-7 kit.

To investigate whether RQA21 posses antimicrobial activity, we tested the peptide against a microbe panel consisting of Gram-positive and Gram-negative bacteria, as well as yeast. The results showed that the peptides were antibacterial in radial diffusion assays (RDA) against the Gram-negative *Escherichia coli *and *Pseudomonas aeruginosa*, the Gram-positive *Bacillus subtilis *and *Staphylococcus aureus*, as well as the fungal isolate *Candida albicans *(Figure 1A). Although variability in the different sensitivities was noted, the zone of inhibition results were lower than those observed for the benchmark antimicrobial peptide LL-37. Interestingly, it was observed that the two clinical Gram-negative isolates (*E. coli *and *P. aeruginosa*) were particularly sensitive to RQA21.

It is well-known that activities of AMPs are dependent of the microenvironment. For example, various chemokines, defensins, as well as LL-37, are partly or completely, antagonized by high salt conditions or the presence of plasma proteins *in vitro *[21,22]. We therefore examined the influence of physiological salt (0.15 M NaCl) on the antimicrobial activity of RQA21 against *E. coli *37.4 using viable count analysis. As shown in Figure 1B, RQA21 efficiently killed *E. coli*, at both 30 and 60 μM in 10 mM Tris buffer. However, at physiological salt conditions the peptide exerted no detectable antimicrobial activity (Figure 1B). This contrasted to LL-37, which was active under both conditions. In order to explore the effect of plasma on the activity of RQA21, different concentrations of citrate plasma (0, 50, 98%) was added to the peptides and activity assayed using RDA (Figure 1C). The activity decreased proportionately with an increase in the plasma concentration, similar effects being observed for the benchmark LL-37.

**Figure 2:**
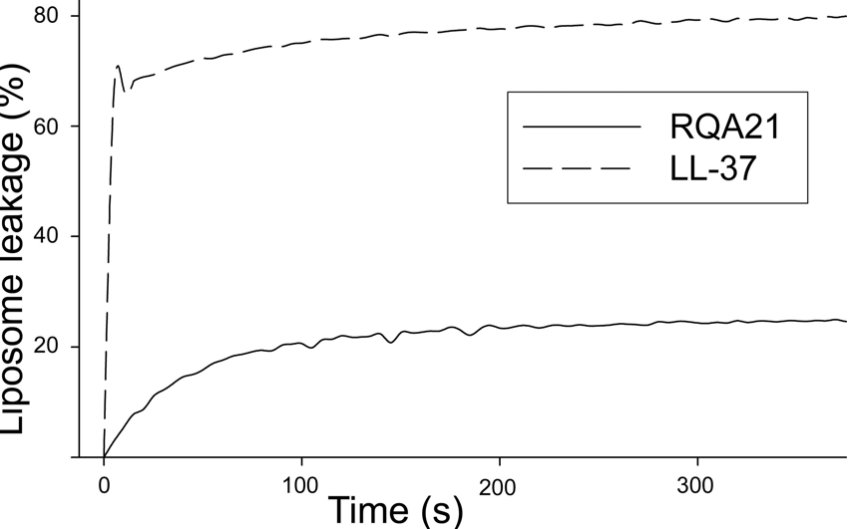
**Effects of RQA21 and LL-37 on liposome leakage kinetics**. The membrane permeabilizing effect of the indicated peptides (at 1 μM) was recorded by measuring fluorescence release of carboxyfluorescein from liposomes.

To shed some further light on RQA21, toxicity studies against human erythrocytes and keratinocytes were performed, with no hemolytic activity detected at doses of 3–60 μM (Figure 1D, left panel). This contrasted to the antimicrobial peptide LL-37, which permeabilised erythrocytes at doses >6 μM. Analogously, RQA21 did not permeabilise human epithelial cells (HaCaT keratinocyte cell line), whereas LL-37 exerted permeabilising activity at doses of 30–60 μM (Figure 1D, right panel). Additionally, RQA21 and LL-37 both permeabilised model lipid membranes at 1 μM. In parallel to the bactericidal results, LL-37 was the more potent of the two peptides in respect to liposome permeabilisation (Figure 2). Kinetic analysis showed that ~80% of the maximal release caused by each peptide occurred within 5–10 minutes for both LL-37 and RQA21 (Figure 2).

## Discussion

The findings in this report, showing that a peptide segment of EC-SOD exerts antimicrobial activities, further underscore that endogenous proteins may harbour cryptic, antimicrobial epitopes. Table 1 exemplifies the diversity of such "non-classical" antimicrobial peptides, originating from diverse proteins and that originate from sequences known to interact with glycosaminoglycans. In the light of this, our findings indicate that a strategy based on selection of peptides of heparin-binding human proteins may prove to be fruitful in the discovery of additional novel sequences as lead candidates for the development of AMPs based on endogenous peptides.

**Table 1:** Examples of "non-classical" antimicrobial peptides, originating from diverse heparin-binding (poly)peptides.

**Origin**	**Sequence**	**Reference**
**Plasma proteins**		
Complement factor C3	LGEACKKVFLDCCNYITELRRQHARAS	[13,14]
High molecular weight kininogen	HKHGHGHGKHKNKGKKNGKH	[15]
		
Fibronectin	QPPRARITGYIIKYEKPG	[17]
Protein C Inhibitor	SEKTLRKWLKMFKKRQLELY	[11]
Histidine-rich glycoprotein	GHHPHGHHPHGHHPHGHHPH	[18,19]
**Extracellular proteins**		
Amphiregulin	LKKNGSCKRGPRTHYGQKAIL	[17]
Heparin-binding EGF-like growth factor	GKRKKKGKGLGKKRDPCLRKYK	[17]
Fibroblast growth factor		
Hepatocyte growth factor	LKIKTKKVNTADQCANRCTRNKGL	
		
Vitronectin	AKKQRFRHRNRKGYR	[11]
PRELP	QPTRRPRPGTGPGRRPRPRPRP	[17]
Laminin chains	SRNLSEIKLLISQARK KDFLSIELFRGRVKV LGTRLRAQSRQRSRPGRWHKVSVRW RLRAQSRQRSRPGRWHKVSVRW	[11]

RQA21 (NH_2_-RQAREHSERKKRRRESECKAA-COOH), is rich in basic amino acids, with a net charge of +6 at pH 7.4, and is also strongly hydrophilic having a low relative hydrophobic moment (0.24, Kyte & Doolittle scale, see . Previous investigations of the structure of the C-terminal part of SOD indicate that the peptide region may assume helical structures, and that arginine side chains take part in the binding to heparin. However, the analysis also suggested that this peptide region exist in an equilibrium between different conformations, where helical as well as random structures are likely to be present [6]. Thus, considering the low hydrophobicity of RQA21, the peptide likely resembles other linear peptides having a low helical content. For example, AMPs derived from growth factors display a low helical content in buffer and in presence of membranes, reflecting their low content of features typical of "classical" helical peptides, such as regularly interspersed hydrophobic residues [17]. Furthermore, studies utilizing ellipsometry, CD, fluorescence spectroscopy, and z-potential measurements on a kininogen-derived antimicrobial peptide, HKH20 (HKHGHGHGKHKNKGKKNGKH) [23] showed that the HKH20 peptide display primarily random coil conformation in buffer and at lipid bilayers, the interactions dominated by electrostatics, as evidenced by strongly reduced adsorption and membrane rupture at high ionic strength [23]. Interestingly, both HKH20 [15] and GKR22 (GKRKKKGKGLGKKRDPCLRKYK) [17], a peptide derived from heparin-binding growth factor, retain antibacterial activity in physiological buffers as well as in plasma. In contrast to these observations, RQA21 lost its antibacterial activity at high salt concentrations, illustrating the well-known fact that cationicity alone is not a sufficient parameter for determining antimicrobial activity of a given peptide, and exemplifying that amphipathicity, as well as hydrophobicity, enabling bacterial membrane interactions, are necessary for activity of many AMPs [1], especially at physiological salt concentrations, where initial electrostatic interactions with anionic cell wall components are diminished by the ionic environment. Clearly, AMPs and other antimicrobial proteins are dependent of the microenvironment, and may be also antagonized the presence of plasma proteins *in vitro *[17,19,21,22]. Nevertheless, there is now convincing evidence that AMPs contribute to enhanced bacterial killing *in vivo*, likely reflecting the necessity of AMP compartmentalization (diminishing toxic effects on nearby eukaryotic cells), presence of ionic microenvironments, or synergism between AMPs, as well as their additional roles as immune modifiers [24].

Bacterial surfaces contain many anionic components, including LPS and anionic lipids of Gram-negative bacteria, as well as teichoic and teichuronic acids of Gram-positive bacteria. Beyond the outer cell surface, AMPs interact with the plasma membrane. Contrasting to eukaryotic membranes, which contain mostly zwitterionic lipids (e.g., phosphatidylcholine), and sterols, bacterial membranes comprise various acidic phospholipids (phosphatidylglycerol, phosphatidylserine and cardiolipin), which confer a negative charge facilitating AMP binding and sometimes also defect formation [25,26]. Furthermore, it has also become increasingly clear that AMP selectivity may also depend on factors such as AMP oligomerisation and preassembly (in solution and membrane) [27]. Clearly, all these factors determine the ultimate activity of a given AMP under specific conditions. Although high AMP activity can sometimes be reached by highly charged and hydrophilic AMPs [15,17], Gram-positive pathogens such as *Staphylococcus aureus *have a relatively low electrostatic surface potential, which may be reduced or even reversed, e.g., by L-lysine modification of phosphatidylglycerol, and D-alanine modification of cell wall teichoic acid, reducing AMP binding to Gram-positive bacteria, respectively [28]. Likely, these mechanisms, together with the high electronegativity of LPS, explain the herein noted higher sensitivity of the two clinical Gram-negative isolates (*E. coli *and *P. aeruginosa*) to the cationic RQA21.

It is well established that SOD serves a key antioxidant role. Mice lacking SOD2 die within days after birth due to massive oxidative stress [29]. In contrast, mice lacking SOD3 (EC-SOD) have a reduced life-span, although not otherwise showing any obvious defects during devlopment and early adulthood [30]. Currently, no data are available on the immune status and infection sensitivity of these mice. Clearly, and as mentioned above, caution should be executed when assigning possible functional roles for peptides based on their *in vitro *activities only. However, our observation, apart from providing a link between heparin-binding peptides and AMP activity *in vitro*, raises the hypothesis that proteolytically generated C-terminal SOD-derived peptides could interact with, and possibly counteract bacteria *in vivo*. Further studies are merited in order to investigate proteolysis of SOD, possible release of C-terminal peptides, and their potential bioactive roles under physiological conditions.

## Competing interests

Martin Malmsten and Artur Schmidtchen are co-founders, and members of the board of DermaGen AB, which develops antimicrobial peptide therapeutics for commercial purposes.

## Authors' contributions

AS and MM conceived the study together, while MP, AS, and MM planned the study and jointly wrote the paper together. MP performed experiments on bacteria. MD performed the eukaryotic assays experiments. MM performed experiments on liposome leakage. All the authors have read and approved the final manuscript.
